# Synthesis, Characterisation and Biological Evaluation of Ampicillin–Chitosan–Polyanion Nanoparticles Produced by Ionic Gelation and Polyelectrolyte Complexation Assisted by High-Intensity Sonication

**DOI:** 10.3390/polym11111758

**Published:** 2019-10-25

**Authors:** Yhors Ciro, John Rojas, Jose Oñate-Garzon, Constain H. Salamanca

**Affiliations:** 1 Department of Pharmacy, School of Pharmaceutical and Food Sciences, University of Antioquia, Medellín 050025, Colombia; ciro-333@hotmail.com (Y.C.); jrojasca@gmail.com (J.R.); 2 Grupo de Investigación en Química y Biotecnología (QUIBIO), Facultad de Ciencias Básicas, Universidad Santiago de Cali, calle 5 No. 62-00, Cali 760035, Colombia; jose.onate00@usc.edu.co; 3 Laboratorio de Diseño y Formulación de Productos Químicos y Derivados, Departamento de Ciencias Farmacéuticas, Facultad de Ciencias Naturales, Universidad ICESI, Calle 18 No. 122-135, Cali 760035, Colombia

**Keywords:** crosslinked chitosan, polyanion, ampicillin, nanoparticles, ultrasound, ionic gelation, polyelectrolyte complexation, antimicrobial activity

## Abstract

Recently, one of the promising strategies to fight sensitive and resistant bacteria, and decrease the morbidity and mortality rates due to non-nosocomial infections, is to use antibiotic-loaded nanoparticles. In this study, ampicillin-loaded chitosan–polyanion nanoparticles were produced through the techniques of ionic gelation and polyelectrolyte complexation assisted by high-intensity sonication, using several crosslinking agents, including phytic acid (non-polymeric polyanion), sodium and potassium salts of poly(maleic acid-*alt*-ethylene) and poly(maleic acid-*alt*-octadecene) (polymeric polyanions). These nanoparticles were analysed and characterised in terms of particle size, polydispersity index, zeta potential and encapsulation efficiency. The stability of these nanosystems was carried out at temperatures of 4 and 40 °C, and the antimicrobial effect was determined by the broth microdilution method using sensitive and resistant *Staphylococcus aureus* strains. The results reveal that most of the nanosystems have sizes <220 nm, positive zeta potential values and a monodisperse population, except for the nanoparticles crosslinked with PAM-18 polyanions. The nanometric systems exhibited adequate stability preventing aggregation and revealed a two-fold increase in antimicrobial activity when compared with free ampicillin. This study demonstrates the potential application of synthesised nanoparticles in the field of medicine, especially for treating infections caused by pathogenic *S. aureus* strains.

## 1. Introduction

Infectious diseases are among the main causes of morbidity and mortality in humans worldwide. In particular, nosocomial infections, caused by a pathogenic strain of *Staphylococcus aureus*, often leads to septicaemia and eventually death [1,2,3,4,5]. These infections are included in the priority list of the World Health Organization due to their resistance against conventional antibiotics, rendering their treatment difficult [6]. This induces high cost in patient care due to testing and the concomitant use of more expensive and nephrotoxic antibiotics, which may increase the chance of mortality to 69% [7]. However, the number of studies searching for new antibiotics has decreased in the last decade [8]. A promising strategy to combat antimicrobial resistance problems deals with the production of nanoparticulate systems loaded with such conventional antimicrobials and, therefore, avoids the biological degradation mediated by such resistant microorganisms [6,9,10,11,12,13,14,15]. Nanoparticles, for instance, could be used as carriers of antimicrobial agents, where the antimicrobial effect could be mediated by different mechanisms, such as (i) direct interaction with the bacterial cell wall; (ii) film formation inhibition; (iii) improvement of the innate and adaptive host immune response; (iv) generation of reactive oxygen species; and (v) induction of intracellular effects [5]. So far, chitosan has been used for the generation of many nanoparticulate systems owing to its biocompatibility, biodegradability and ability to modulate the release of active compounds. For instance, Ngan and collaborators developed amoxicillin-loaded chitosan nanoparticles, which improve antimicrobial activity [16]. Another study revealed the prominent effect of sodium phytate–chitosan nanoparticles against Gram-positive and Gram-negative bacteria [17]. Ibrahim and collaborators proved that chitosan nanoparticles exhibit higher antibacterial activity against Gram-positive bacteria than Gram-negative bacteria [18].

Currently, there are a vast number of reports on the development of chitosan nanoparticles using different techniques, where ionic gelation [19,20,21,22,23,24,25] and polyelectrolyte complexation [26,27,28,29,30] are the most widely used. However, to date there are very few studies focussed on evaluating the antimicrobial effect provided by drugs such as ampicillin loaded in chitosan nanoparticles cross-linked with different polymeric anionic agents. Therefore, the goals of this study are (i) to produce and characterise the ampicillin-loaded chitosan–polyanion nanoparticles obtained by ionic gelation and polyelectrolyte complexation assisted by high-intensity sonication, and (ii) evaluate their antimicrobial activity on *S. aureus* strains having different antimicrobial-resistance degrees.

## 2. Materials and Methods

### 2.1. Materials

Commercial chitosan with a deacetylation degree around 75% (lot STBF8219V) was purchased from Sigma-Aldrich Co. (St. Louis, MO, USA). The anionic polyelectrolytes corresponding to the sodium and potassium salts of poly(maleic acid-*alt*-ethylene) (PAM-2Na or PAM-2K) and poly(maleic acid-*alt*-octadecene) (PAM-18Na or PAM-18K) previously synthesized and characterized [31,32], were provided by the Laboratory of Design and Formulation of Chemical Products from Icesi University (Cali, Colombia). Such anionic polymers were utilized as received. The phytic acid (PA) solution (MKCB0619V) was purchased from Sigma Aldrich Co (St. Louis, MO, United States). Sodium hydroxide (lot B1315798639) and acetic acid (lot K41575763) were obtained from Merck (Darmstadt, Germany). Ampicillin trihydrate (Amp) was provided by Tecnoquimicas S.A. (Cali, Colombia). *Staphylococcus aureus* strains (ATCC25923, ATCC29213 and ATCC43300) were obtained from Microbiologics Inc.^©^ (St. Cloud, MN, USA) and were reconstituted according to the supplier instructions.

### 2.2. Production and Characterisation of Highly Deacetylated Chitosan 

The deacetylation reaction was carried out using a focused microwave apparatus with a power output of 600 Watts adjustable in ten increments, operated at a 10% power (Samsung, Model MW 630 WA; Bueng, Tailandia, dimensions: 289 mm × 179 mm × 326 mm) for 2 h, using additive cycles of 5 min. Approximately, 50 mL of a 10% (*w*/*v*) chitosan dispersions (made with 10N NaOH) was poured into a 500 mL round-bottom flask, coupled with a 300-mm-long spiral reflux condenser through an aperture on top of the microwave apparatus. Subsequently, the suspension was neutralized with 6N HCl, vacuum filtered and dialyzed using a cellulose membrane, with a cut-off of 12 kDa, and deionized water until a conductivity ~2 µS/cm was reached. The suspension was then lyophilised at −45 °C and 0.04 bar (Eyela FDU-1100, Rikakikai Co., Tokyo, Japan). Subsequently, the viscosity-average molecular weight of chitosan and deacetylated chitosan was determined by intrinsic viscosimetry using solutions having concentrations between 0.01 and 0.09 g/dL at 25 °C. The dispersion medium employed was composed of a mixture of 0.1 M acetic acid/0.2 M sodium chloride. The Mark–Houwink–Sakurada equation was used to calculate the polymer molecular weight as follows:

(1) $$[\eta ]\;=\;k*{M_{v}}^{\alpha }$$

where [*η*] and *M*_v_ correspond to the intrinsic viscosity and viscosity-average molecular weight of the polymer, and *k* and *α* are constants related to the solvent and the 3D conformation (linear or branched) of the polymer and have values of 1.81 × 10^−5^ dL/g and 0.93, respectively [33]. On the other hand, the deacetylation degree was determinate by IR spectroscopy. In this case, the IR spectrum of the polymers were recorded between 400 and 4000 cm^−1^ on a FT-IR spectrophotometer (Nicolet 6700, Thermo Scientific, Waltham, WA, USA) at a resolution of 4 cm^−1^ and 32 scans. Approximately 10 mg of polymer was mixed with ~200 mg of KBr (previously dried at 120 °C for 3h) with an agate mortar and pestle. The mixture was then compressed in a hydraulic press (060804 Compac, Indemec, Itagui, Colombia) using a 13 mm flat-faced punches and die tooling and a dwell time of 1 min. The degree of acetylation (*DA*) was found by taking the ratio of the FT-IR absorbance bands obtained at 1650 and 3450 cm^−1^, according to the Baxter equation:

(2) $$DA\;(\%)\;=\;\frac{A_{1650}}{A_{3450}}*115$$

where *DA* corresponds to the degree of acetylation, 115 is the ratio of the molecular weight of the *N*-acetyl-glucosamine and *N*-glucosamine subunits, and A_1650_ and A_3450_ correspond to the type I amide and hydroxyl stretching bands, respectively [34,35]. 

### 2.3. Preparation of Nanoparticulate Systems

**Figure 1:**
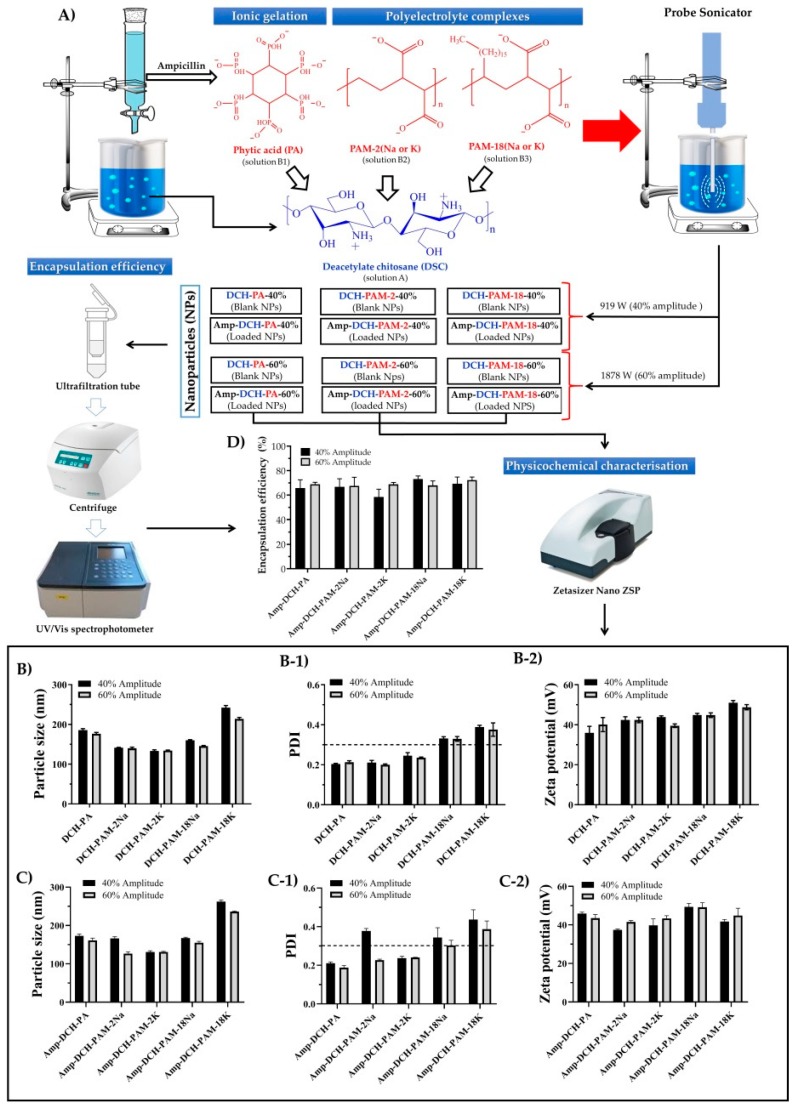
(**A**) Schematic of the formation of chitosan–polyanion nanoparticles unloaded and loaded with ampicillin, using high-intensity ultrasounds. (**B**) Characterisation of particle size, polydispersity index and zeta potential for chitosan–polyanion nanoparticles without ampicillin (blank NPs). (**C**) Characterization of particle size, polydispersity index and zeta potential for chitosan–polyanion nanoparticles loaded with ampicillin. (**D**) Encapsulation efficiency for ampicillin-loaded chitosan–polyanion nanoparticles.

A 3 mg/mL chitosan solution (in 1% acetic acid, *v*/*v*) with a pH of 3.5 was prepared and labelled as solution A. At the same time and independently, several aqueous solutions corresponding to ampicillin (5 mg/mL), phytic acid (0.5 mg/mL), PAM-2Na (0.5 mg/mL), PAM18K (0.5 mg/mL), PAM18Na (0.5 mg/mL) and PAM-18K (0.5 mg/mL) were prepared. Subsequently, five mixtures between the ampicillin solution with each polyanion solution were made. Particularly, 1.0 mL of ampicillin solution and 11.0 mL of phytic acid solution (solution B1), 1.0 mL of ampicillin solution and 15.0 mL of PAM-2 solutions (solution B2) and 1.0 mL of ampicillin solution and 15.0 mL of PAM-18 solution (solution B3) were mixed. Subsequently, each solution “B” was poured into solution A, which remained under constant magnetic stirring at 800 rpm and 25 ± 1 °C. Such mixtures were left under constant stirring for 10 additional minutes in order to generate the complexes by ionic association. Thus, the ampicillin–chitosan–phytic acid complexes were formed by ionic gelation, whereas the ampicillin–chitosan–PAM-2 and ampicillin–chitosan–PAM-18 complexes were formed by polyelectrolyte complexation. Once the ionic association complexes were formed, their sizes were reduced employing a probe sonicator. Particularly, a 4.0 mL aliquot of each complex dispersion was taken and subjected to ultrasonic treatment using an ultrasonic probe (CL-18, tip 4422, diameter of 3 mm). Pulses of 30 s each followed by a 30 s resting time was employed for a total treatment of 5 min. An energy intensity of 919 W and 1878 W corresponding to a 40% and 60% amplitude were employed. On the other hand, blank nanoparticles were created following the same procedure where the solutions B had no ampicillin (Figure 1A).

### 2.4. Physicochemical Characterisation of the Nanoparticles

#### 2.4.1. Particle Size, Polydispersity Index (*PDI*) and Zeta Potential Analyses

These analyses were determined using a Zetasizer nano ZSP (Malvern Instrument, Worcestershire, United Kingdom) equipped with a red He/Ne laser (633 nm). Particle size was measured using a dynamic light scattering (DLS) with a scattered angle of 173° at 25 °C, and a quartz flow cell (ZEN0023), whereas the zeta potential was measured using a disposable folded capillary cell (DTS1070). This instrument reports the particle size as the z-average diameter, and PDI ranging from 0 to 1 corresponding to monodispersed and very broad distributions, respectively. All the nanoparticles were dispersed in ultra-pure water employing an ~1:100 *v*/*v* dilution factor. All measurements were performed in triplicate and reported as the mean ± standard deviation.

#### 2.4.2. Encapsulation Efficiency (*EE*)

The *EE* of ampicillin was assessed by employing the ultrafiltration/centrifugation technique. An aliquot of each nanoparticulate suspension was poured into an ultrafiltration tube (VWR, Modified PES 10 kDa, 500 µL) and centrifuged (MIKRO 185, Hettich Lab Technology, Tuttlingen, Germany) at 10,000 rpm for 6 min. Subsequently, 200 µL of the filtrate solution was taken and mixed with 800 µL of a 200 µg/mL ampicillin solution. The absorbance of the resulting mixture was measured on a UV/Vis spectrophotometer (UV-1800, Shimadzu, Milton Keynes, UK) at 262 nm. The amount of ampicillin was determined by interpolation from a calibration curve built at concentrations of 20, 50, 80, 100, 150, 200 and 300 µg/mL using a mixture of water/1% (*v*/*v*) acetic acid (80:20) as solvent. The quantity of the ampicillin loaded inside the nanoparticles was calculated using the following expression:

(3) $$EE\;=\;\frac{Q_{t}-\;Q_{s}}{Q_{t}}\;*\;100\%$$

where *Q*_t_ and *Q*_s_ correspond to the total amount of ampicillin and the amount of ampicillin found in the filtrate, respectively.

### 2.5. Stability of the Nanoparticle Systems 

The stability of the nanoparticulate systems was evaluated in chambers maintained at 4 °C and 40 °C for 5 days. Approximately 2 mL of nanoparticulate suspensions were stored, and the physicochemical parameters, such as the zeta potential, particle size and polydispersity index, were measured at the initial and final stage of the experiment, as previously described in Section 2.4.1.

### 2.6. Antimicrobial Effect of the Nanoparticles

The antimicrobial effect of ampicillin, blank nanoparticles and ampicillin-loaded nanoparticles were determined by the broth microdilution method according to the guidelines of the Clinical and Laboratory Standards [36]. The bacteria were inoculated in a Mueller–Hinton broth (MHB) at 37 °C for 24 h and then diluted with MHB broth until an optical density (absorbance at 625 nm) of 0.1 (~1 × 10^−8^ CFU/mL) was reached. Subsequently, a 1/1000 dilution factor was employed (~1 × 10^−5^ UFC/mL) for the tests. Particularly, 50 µL of this bacterial culture was incubated for 18–20 h in 96-well plates at 37 °C along with 50 µL of each nanoparticle sample. A two-fold serial dilutions ranging from 0.008 to 256 µg/mL were used for each sample. A saline phosphate buffer was used as a negative control. The minimal inhibitory concentration (MIC) was visually determined after incubation. The test was conducted in triplicate.

### 2.7. Statistical Analysis

Data were tabulated and analysed using the Minitab^®^ v. 17 software (Minitab^®^ Inc., State College, PA, USA). Statistical comparisons were made employing the ANOVA test, where the effect of sonication amplitude and polyanion type on the particles size, PDI, zeta potential and encapsulation efficiency were evaluated. The Tukey post-hoc test was utilized to determine significant differences between the independent groups. A confidence level of 95% was adopted and data were expressed as the mean ± standard deviation.

## 3. Results and Discussion

### 3.1. Production and Characterisation of a Highly Deacetylated Chitosan

The alkaline modification of the commercial chitosan was carried out in order to obtain a new chitosan having a greater deacetylation degree, thus achieving a cationic polyelectrolyte with a better ability to generate charges in the main polymer chain, favouring the electrostatic interaction processes required for the nanoparticle formation. In this scenario, the comparison between the IR absorption bands (Figure S1 is shown in the Supporting Material file) for the commercial chitosan and modified chitosan displayed a particular change in the deacetylation degree from 75% to more than 90%, which is, in fact, the expected result for such modification [37]. Meanwhile, the molecular weight decreased from 680 to 477 kDa as a consequence of a mild depolymerisation, which took place simultaneously [38].

### 3.2. Production and Characterisation of Nanoparticulate Systems

The results from the physicochemical characterisation of the chitosan–polyanion nanoparticles (i.e., particle size, PDI, zeta potential and encapsulation efficiency) obtained by ionic gelation and polyelectrolyte complexation, both assisted with high-intensity ultrasounds are shown in Figure 1. 

On the other hand, the data resulted from the statistical analysis are summarized in Table 1.

Table 1 shows that the sonication amplitude and the type of polyanion affect the physicochemical features of the nanoparticles in different ways. Therefore, the discussion of the results is carried out by analysing each one of these characteristics separately.

#### 3.2.1. Particle Size

According to the statistical analysis shown in Table 1, it was found that the sonication amplitude affected the ampicillin-loaded and blank nanoparticles altogether, where the higher energy treatment led to a greater reduction in particle size. Likewise, it was observed that the type of polyanion also influenced such a physicochemical property, being the PAM-18K polyanion, the one that rendered the largest particle size, followed by phytic acid and PAM-18Na. Conversely, the PAM-2Na and PAM-2K produced the smaller sizes. All these results were expected since during the sonication process, high-energy acoustic shock waves are formed, which in turn disintegrate and fractionate the nanoparticles, especially in those areas where the interactions between chitosan and polyanions are weaker. However, in order to understand these particular phenomena, it is necessary to analyse the different types of interactions generated between chitosan and each type of anionic polyelectrolyte.

**Figure 2:**
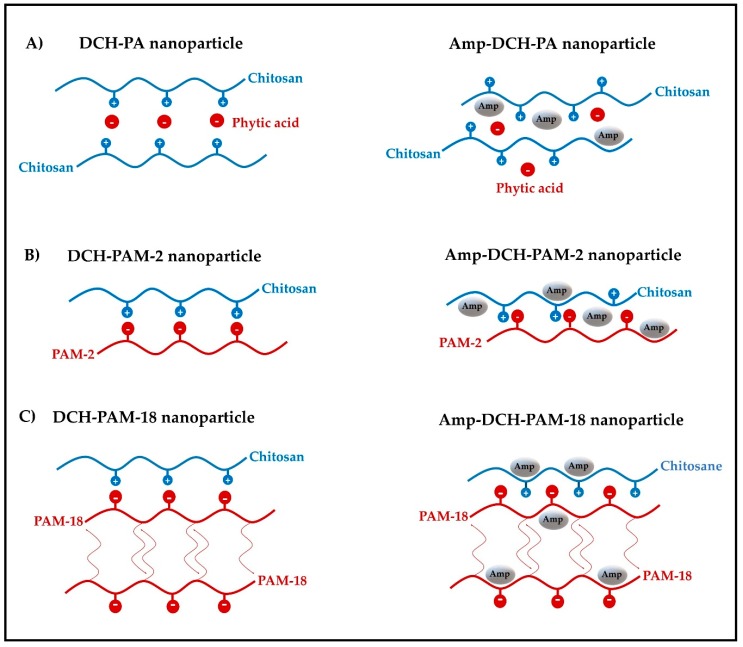
Schematic of the formation of chitosan–polyanion nanoparticles in blank (**left**) and ampicillin-loaded (**right**) nanoparticles. (**A**) Nanosystem formed with phytic acid. (**B**) Nanosystem formed with PAM-2. (**C**) Nanosystem formed with PAM-18.

In the case of nanoparticles formed by chitosan and phytic acid, it was found that the increase in sonication amplitude led to a slight decrease in size of both nanoparticles (Figure 1B,C), ranging from 185.6 ± 3.7 nm to 176.7 ± 3.1 nm for blank nanoparticles (DCH-PA) and from 173.1 ± 4.4 nm to 161.2 ± 6.0 nm for ampicillin-loaded nanoparticles (Amp-DCH-PA). Likewise, it was observed that the antibiotic led to a slight decrease in particle size and, therefore, such nanoparticles tend to create more compact reticulated structures [39,40,41]. Therefore, this phenomena can be explained considering the chemical structure of ampicillin [42], which has several polar moieties (i.e., carboxylic acid, amide and amine), which can form multiple hydrogen bonds and ion-dipole interactions between chitosan and phytic acid (Figure 2A).

Likewise, the nanoparticulate systems produced with chitosan and the sodium and potassium salts of poly(maleic acid-alt-ethylene) (DCH-PAM-2 and Amp-DCH-PAM-2) showed that the increase in sonication amplitude led to a slight decrease in the nanoparticles size (Figure 1B,C), and these values ranged from 130 nm to 140 nm, except for the Amp-DCH-PAM-2Na system, which was higher (166.2 ± 4.3 nm). This series of nanoparticles showed the smallest particle size, suggesting that these are formed by very neat structures. This result is explained considering the high molecular weight of the PAM-2 polyanion (~100 kDa), which leads to a greater inter-polymeric electrostatic attraction, where the PAM-2 migrates to the chitosan interface forming very compact aggregates (polyelectrolyte complexation) [43,44,45,46,47]. Likewise, the presence of ampicillin also led to a decrease in particle size, as explained previously for the nanoparticles produced with phytic acid (Figure 2B). 

On the other hand, a similar synergistic effect of the decrease in particle size given by the sonication amplitude and ampicillin was also observed for the nanoparticles formed with chitosan and the salts of the poly(maleic acid-alt-octadecene). However, the nanoparticles produced with the PAM-18 polymer and especially with the potassium salt (Figure 1B,C) showed the highest particle size values. In this case, a marked effect of the counterion was observed, where the nanosystems formed with PAM-18Na showed values between 145 nm and 167 nm, whereas the PAM-18K exhibited values between 214 nm and 262 nm. This effect can be explained by the size and ionic mobility of the counterion, where potassium ions tend to be more tightly bounded to the polyanion, (tight ion pair) and thus the electrostatic interactions given between chitosan and the polyanion are less cohesive than those shown by the sodium salt. Further, such PAM-18 polymers have an alkylic side chain of 18 carbon atoms and, therefore, once they are dispersed in aqueous media, these polymers tends to spontaneously acquire a random coil configuration and form inter-polymer aggregates [48,49,50], avoiding the effect of hydrophobic repulsion, resulting in high particle sizes (Figure 2C).

#### 3.2.2. Polydispersity

Table 1 shows that the sonication amplitude only affects the nanoparticles loaded with ampicillin, where the greater sonication amplitude produces less polydispersity. Conversely, the polyanion type does affect this parameter in different ways. For instance, the nanoparticles formed with the sodium and potassium salts of PAM-18 (Figure 1(B-1,C-1)), exhibited the highest values of PDI (>0.3), whereas those produced by phytic acid and PAM-2 rendered the lowest PDI values (≤0.3).

In the case blank nanoparticles produced with PAM-18 at an amplitude of 40%, showed PDI values of 0.332 ± 0.008 (PAM-18Na) and 0.389 ± 0.009 (PAM-18K), whereas that produced at an amplitude of 60% showed PDI values of 0.330 ± 0.012 and 0.376 ± 0.033 for PAM-18Na and PAM-18K, respectively. Likewise, the nanoparticles loaded with ampicillin at an amplitude of 40% showed PDI values of 0.344 ± 0.051 (PAM-18Na) and 0.437 ± 0.051 (PAM-18K), while those made at an amplitude of 60% showed PDI values of 0.303 ± 0.027 and 0.387 ± 0.043 for PAM-18Na and PAM-18K, respectively. These results are consistent with those obtained for particle size, where the sonication amplitude and the presence of ampicillin lead to more compact and less polydispersed nanoparticulate systems. Likewise, polydispersity increases when the PAM-18, having the potassium counterion, are used, proving that these polyanions lead to the formation of less organized polyelectrolytic complexes (Figure 2C). On the other hand, the nanosystems formed with PAM-2 showed PDI values <0.3, except for the Amp-DCH-PAM2–40% system, which had a PDI value of 0.378. However, the application of greater ultrasonic energy led to a PDI decrease of 0.227, indicating that such a process effectively disintegrates and reorganizes the nanoparticles leading to a more homogeneous size population. Further, the nanoparticles formed with phytic acid had a less polydisperse population, since this polyanion is non-polymeric, and hence, small enough to be located between the chitosan chains, forming more homogeneous crosslinked structures (Figure 2A).

#### 3.2.3. Zeta Potential 

According to the statistical results listed in Table 1, the sonication amplitude does not affect this physicochemical property. However, the type of polyanion employed leads to different values without any specific trend. Nevertheless, the most striking result is the fact that zeta potential values were always positive regardless of the polyanion used and the presence of ampicillin. These results can be explained, considering the macromolecular nature of chitosan having a high molecular weight (~477 kDa) with predominant amino-type moieties that are protonated in acidic media. In addition, the amount of chitosan for the production of the nanoparticles was 10 times larger with respect to that of the polyanion and, therefore, such nanoparticulate systems always have a predominant positive charge at their interface. 

Further, phytic acid leads to zeta potential values between +36.0 mV and +40.1 mV for blank nanoparticles, whereas values ranging from +45.9 mV to +43.5 mV were obtained for ampicillin-loaded nanoparticles (Figure 1(B-2,C-2)). Likewise, the nanoparticles produced with the PAM-2 polyanion rendered zeta potential values between +39.5 mV and +43.8 mV for blank nanoparticles, whereas the loaded nanoparticles showed valued ranging from +37.4 mV to + 43.4 mV. On the other hand, the PAM-18 polyanion rendered nanoparticles having zeta potential values ranging from +44.8 mV to +51.0 mV and +41.7 mV to +49.4 mV for blank and loaded nanoparticles, respectively. This result is coherent, since such material is amphiphilic in nature, allowing for the formation of inter-polymer aggregates between the side chains and not exclusively with the ionic interface of chitosan (Figure 2C).

#### 3.2.4. Encapsulation Efficiency

Table 1 showed that the sonication amplitude and the polyanion type did not have a significantly effect on this property, achieving association efficiencies from 60% to 70%. This means that the antibiotic is effectively associated within the nanoparticles possibly in several ways. In the case of nanoparticles formed with phytic acid, it is plausible that ampicillin is trapped within the reticules formed between the chitosan chains and the polyanion. Conversely, the nanosystems formed with PAM-2 could entrap the ampicillin within the reticules of the polyelectrolyte complex, as well as adsorption at the polymer–water interfacial areas. Further, it is expected that the same ampicillin association phenomena as described for PAM-2 along with solubilisation of the antibiotic within the polymeric alkyl aggregates is true in the case of PAM-18. Moreover, other studies conducted on tripolyphosphate–chitosan nanoparticles produced by electrospraying have shown entrapment efficiencies around 80% for sodium ampicillin due to a bigger particle size effect [51]. Further, Wang et al. synthetized superparamagnetic chitosan microparticles having an association efficiency of 55% for sodium ampicillin [52].

### 3.3. Stability of the Nanoparticulate Systems 

**Figure 3:**
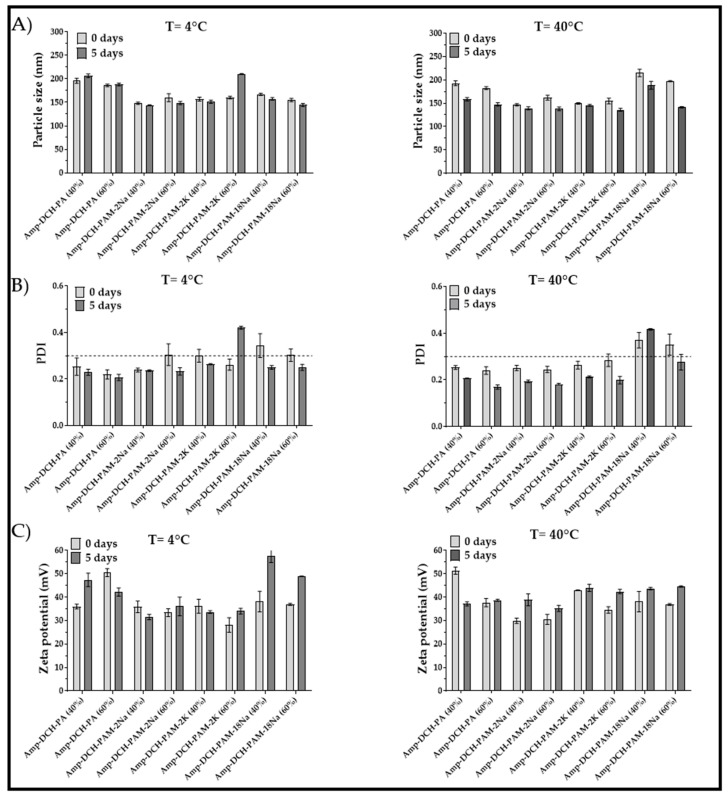
Stress studies conducted at 4 °C and 40 °C for ampicillin-loaded nanoparticulate systems: (**A**) particle size, (**B**) polydispersity and (**C**) zeta potential.

Results from the stability studies conducted at 4 and 40 °C are presented in Figure 3. Particle size did not change significantly upon storage at 4 °C, except for the PAM-2K system obtained at 60% amplitude, which showed a 30% and 60% increase in size and PDI, respectively. Furthermore, the PDI decreased for most nanoparticulate systems, suggesting that a reduction in temperature increased the degree of crosslinking between deacetylated chitosan and the anionic agent, generating more homogeneous sizes. On the contrary, these nanoparticles suffered from changes in the zeta potential due to rearrangement of polymers into nanoaggregates, exposing the amine groups of deacetylated chitosan. On the other hand, the thermal stress studies conducted at 40 °C showed a reduction of size and PDI for all the nanoparticulate systems. This effect might be explained by the rearrangement of the polymer chains, leading to a higher crosslinking effect. This phenomenon is common in dispersed colloidal systems having an excess of chitosan [53]. Further, the thermal treatment caused slight changes in the zeta potential, and all values were larger than +30 mV, indicating good electrostatic repulsive forces. There was no evidence of oxidation as reported in other studies [51]. The above results reveal that these nanoparticulate systems are stable over time and thus they did not exhibit any significant change of the physicochemical properties.

### 3.4. Antimicrobial Effect of the Nanoparticles

**Figure 4:**
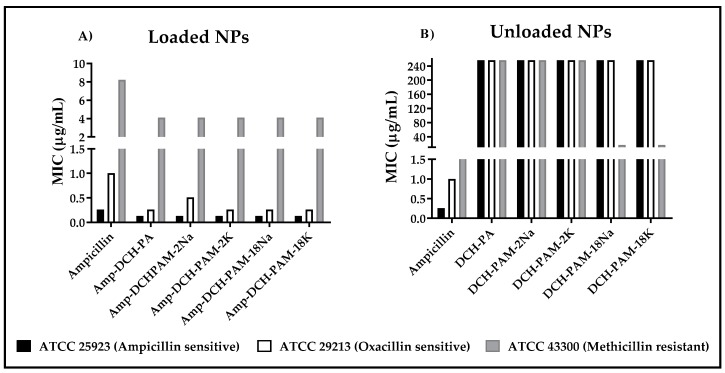
Minimum inhibitory concentration for (**A**) ampicillin–chitosan–polyanion nanoparticles and (**B**) blank chitosan–polyanion nanoparticles.

In this case, the biological evaluation was evaluated only for the systems obtained with the maximum amplitude of sonication, because these showed an appropriate stability and efficiency of encapsulation, as well as the smaller sizes and polydispersities. The antibacterial effect of loaded nanoparticles (Amp-DCH-PA, Amp-DCH-PAM-2 and Amp-DCH-PAM-18) and blank nanoparticles (DCH-PA, DCH-PAM-2 and DCH-PAM-18) over different *S. aureus* strains are depicted in Figure 4A,B. A 50% MIC increase was observed once ampicillin was encapsulated within these complexes, independent of the resistance degree. In the ampicillin-sensitive strain (ATCC 25923), the MIC for the free ampicillin and encapsulated antibiotic was 0.26 µg/mL and 0.13 µg/mL, respectively. This outcome suggests a synergistic effect between the ampicillin and the nanoparticulate carrier. It has been reported that both chitosan and PAM-18 polymer, when combined with b-lactam drugs, forming different types of nanostructures systems, have shown antibacterial activity against *S. aureus* with different degrees of resistance [13,14,15]. Further, the positively charged chitosan is preliminarily attracted to the lipoteichoic acid found on the surface of Gram-positive bacteria (LTA) by electrostatic interactions [54]. This interaction takes place more specifically in the outer leaflet of the cytoplasmic membrane of *S. aureus* [55]. As a result, this interaction disturbs the cell membrane homeostasis affecting bacterial viability. In fact, Halder and collaborators reported that agents having a net positive charge could induce alterations in the zeta potential of the cell membrane enhancing its permeability [56].

On the other hand, the MIC obtained for the free and encapsulated ampicillin applied on ampicillin-resistant and oxacillin-sensitive *S. aureus* (ATCC 29213) was 1.0 µg/mL and 0.26–0.51 µg/mL, respectively. These values were higher than that exhibited by the ATCC 25923 strain. This is explained by its ability to secrete *β*-Lactamase as a resistance mechanism. Results also confirmed the protective effect once ampicillin was encapsulated as reported previously [13,57].

Interestingly, *S. aureus* (MRSA) exhibited the highest ampicillin resistance due to an additional mechanism as compared to the other two strains (ATCC 25923 and ATCC 29213). The former strain exhibits a modified penicillin-binding protein (PBP2a) [58]. Therefore, the MIC for this strain was 8 µg/mL and 4 µg/mL for the free ampicillin and ampicillin-loaded nanoparticles. Remarkably, either the DCH-PAM-18K and DCH-PAM-18Na nanoparticles were the only systems exhibiting antimicrobial activity in the absence of ampicillin. Thus, DCH-PAM-18 nanoparticles (Figure 4b) exhibited a 4-fold lower antibacterial activity than that exhibited by ampicillin-loaded nanoparticles. These results suggest that deacetylated chitosan and PAM-18 polyanions essentially interfere with the metabolic pathway of the PBP2a protein, since the systems having no ampicillin do not show antimicrobial effect against the other *S. aureus* strains. The antibacterial activity of chitosan has been corroborated against resistant microorganisms [59]. Eom et al. [60] established that ferulic acid–chitosan conjugates have the ability to decrease the expression of the *mecA* gene which, in turn, is responsible for the PBP2a expression. On the other hand, *S. aureus* has a unique membrane composed of anionic phospholipids, such as phosphatidylglycerol and cardiolipin [61], which are electrostatically attracted to the cationic chitosan. Further, it has been demonstrated that cationic compounds have the ability to create phospholipid domains within the cell membrane [62], which could interfere with the PBP2a oligomerization, deactivating the methicillin resistance [63]. Therefore, these results suggest two approaches responsible for the antibacterial activity of these polymers and a decrease in MRSA resistance: (i) the cationic system is electrostatically attracted to LTA. The polymer must be dissociated from the system in order to get across the cell wall pores having sizes ranging from 2.06 to 3 nm [64] and, thus, disrupt the cell membrane [65] or targets the mRNA for the *mecA* gene [60]; and (ii) the polymer enter into the cell wall and interacts with the PBP2a protein preventing substrates binding onto the cell wall [66]. In a study conducted by Fuda and collaborators, it was determined that some cephalosporins have the ability to acylate the PBP2a protein, generating a complex which prevents the recovery of protein activity [67].

## 4. Conclusions

Except for PAM-18K, all the anionic crosslinking agents exhibited an adequate capacity to interact with deacetylated chitosan, forming nanoparticles with sizes smaller than 220 nm and a very low polydispersity. The ampicillin–chitosan–polyanion nanoparticles produced by high-intensity sonication and ionic gelation exhibited a high encapsulation efficiency between about 60% and 70%. These nanosystems had positive zeta potential values ranging from +35 to 45 mV, awarding them with a suitable stability preventing aggregation. These novel nanosystems produced a two-fold enhancement of antimicrobial activity against sensitive and resistant strains of *S. aureus*, showing a potential application in medicine, and specifically for the development of new strategies to combat drug resistance.

## Figures and Tables

**Table 1:** Results from the post-hoc Tukey test assessing the effect of sonication amplitude and polyanion type on the physicochemical characteristics of chitosan–polyanion nanoparticles (i.e., particle size, PDI, zeta potential and encapsulation efficiency).

**Factor**	**Ampicillin-Unloaded Nanoparticles**						**Ampicillin-Loaded Nanoparticles**					
	**Particle Size (nm)**						**Particle Size (nm)**					
	**Level**	**Average**	**Group**				**Level**	**Average**	**Group**			
Sonication Amplitude	40	172.6	A				40	179.8	A			
	60	162.1		B			60	162.0		B		
Type of polyanion	PAM-18K	228.1	A				PAM-18K	249.2	A			
	Phytic Acid	181.2		B			Phytic Acid	167.1		B		
	PAM-18Na	153.0			C		PAM-18Na	161.1		B		
	PAM-2Na	140.7				D	PAM-2Na	146.5			C	
	PAM-2K	133.8				D	PAM-2K	130.7				D
	**Polydispersity**						**Polydispersity**					
**Factor**	**Level**	**Average**	**Group**				**Level**	**Average**	**Group**			
Sonication Amplitude	40	0.276	A				40	0.321	A			
	60	0.271	A				60	0.269		B		
Type of Polyanion	PAM-18K	0.382	A				PAM-18K	0.412	A			
	PAM-18Na	0.331		B			PAM-18Na	0.323		B		
	PAM-2K	0.240			C		PAM-2Na	0.303		B	C	
	Phytic Acid	0.208				D	PAM-2K	0.239			C	D
	PAM-2Na	0.206				D	Phytic Acid	0.199				D
	**Zeta Potential (mV)**						**Zeta Potential (mV)**					
**Factor**	**Level**	**Average**	**Group**				**Level**	**Average**	**Group**			
Sonication Amplitude	40	+43.6	A				60	+44.5	A			
	60	+43.1	A				40	+42.8	A			
Type of Polyanion	PAM-18K	+49.9	A				PAM-18Na	+49.2	A			
	PAM-18Na	+44.8		B			Phytic Acid	+44.7		B		
	PAM-2Na	+42.4		B			PAM-18K	+43.3		B	C	
	PAM-2K	+41.7		B	C		PAM-2K	+41.6		B	C	
	Phytic Acid	+38.0			C		PAM-2Na	+39.5			C	
	**Encapsulation Efficiency**						**Encapsulation Efficiency (%)**					
**Factor**	**Level**	**Average**	**Group**				**Level**	**Average**	**Group**			
Sonication Amplitude	-	-	-	-	-	-	60	69.1	A			
	-	-	-	-	-	-	40	66.7	A			
Type of Polyanion	-	-	-	-	-		PAM-18K	70.8	A			
	-	-	-	-	-		PAM-18Na	70.6	A			
	-	-	-	-	-		Phytic Acid	67.3	A			
	-	-	-	-	-		PAM-2Na	67.2	A			
	-	-	-	-	-		PAM-2K	63.7	A			

The means that do not share a letter are significantly different.

## Supplementary Materials

The following are available online at https://www.mdpi.com/2073-4360/11/11/1758/s1, Figure S1: Infrared spectrum for commercial chitosan and alkali-processed chitosan with a degree of deceleration >90% (chitosan deacetyl).
