# Supplementary material for: Synthesis, Characterisation and Biological Evaluation of Ampicillin–Chitosan–Polyanion Nanoparticles Produced by Ionic Gelation and Polyelectrolyte Complexation Assisted by High-Intensity Sonication
Source: Polymers (Basel). 2019 Oct 25;11(11):1758. doi: 10.3390/polym11111758 (PMC6918291; doi:10.3390/polym11111758)
Supplement: Supplementary file 1 [file polymers-11-01758-s001.pdf]

Support material

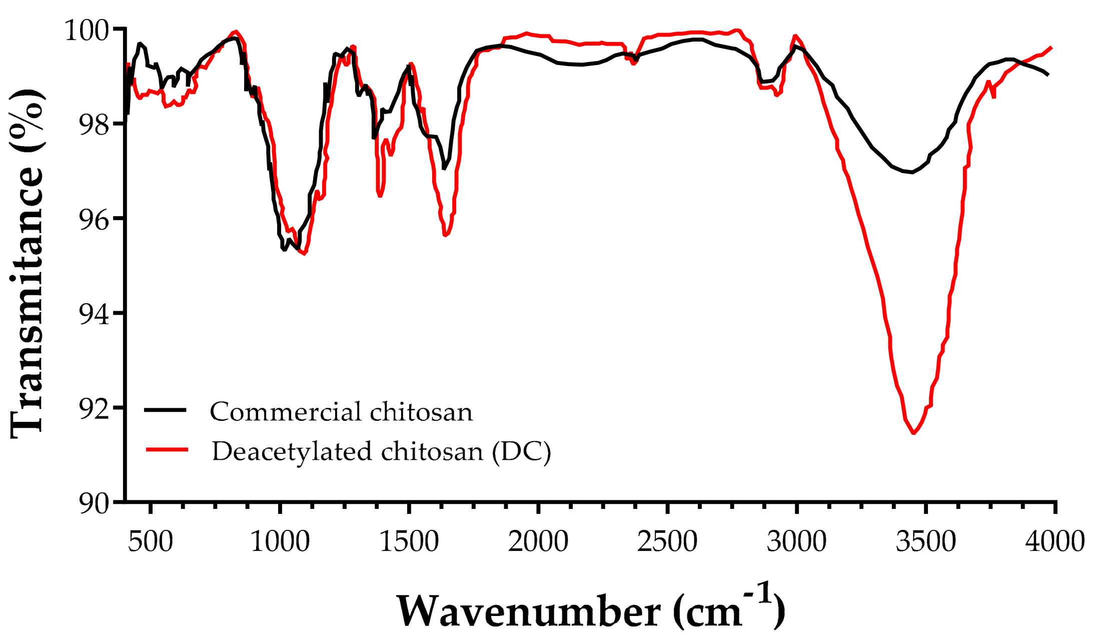

Figure S1. Infrared spectrum for commercial chitosan and alkali-processed chitosan with a degree of deceleration> 90% (chitosan deacetyl)
